# Supplementary material for: Combination radiation and αPD-L1 enhance tumor control by stimulating CD8+ PD-1+ TCF-1+ T cells in the tumor-draining lymph node
Source: Nat Commun. 2025 Apr 14;16:3522. doi: 10.1038/s41467-025-58510-1 (PMC11997041; doi:10.1038/s41467-025-58510-1)
Supplement: Supplementary file 1 — Supplementary Information [file 41467_2025_58510_MOESM1_ESM.pdf]

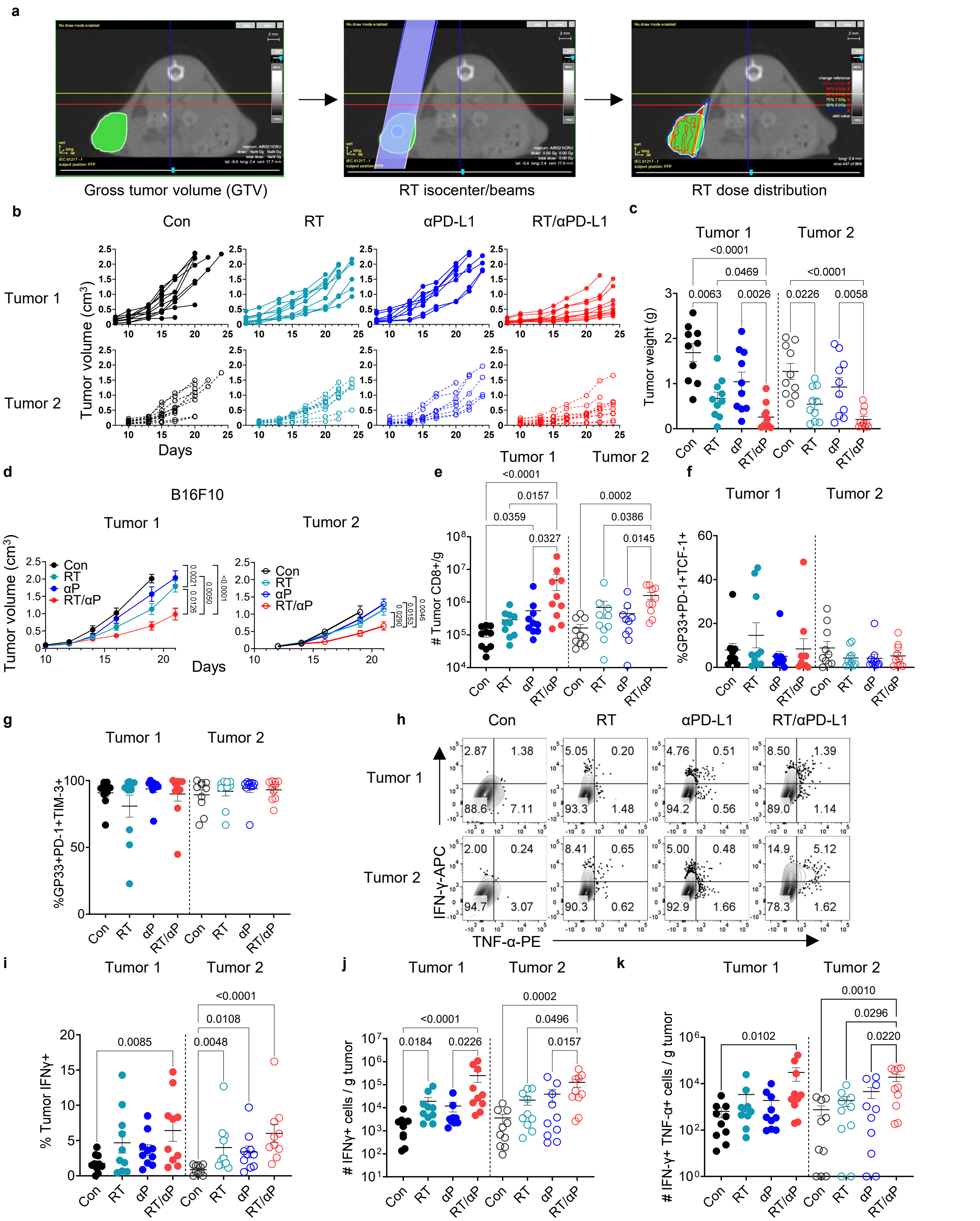

Supplementary Figure 1. RT + αPD-L1 promote tumor control and an increase in intra-tumoral IFNγ+ CD8+ T cells, related to Figure 1. (**a**) Representative images showing tumor GTV, RT isocenter, beams, and dose distribution with isodose lines. (**b**) Tumor growth kinetics under different treatment conditions for B16F10GP. Data reflect 2 separate experiments combined (Con n=8, RT n=9, αPD-L1 n=8, RT/αPD-L1 n=10 total). (**c**) Tumor weights under different treatment conditions. Data are presented as mean values +/- SEM. Data reflect combined data from 2 separate experiments (n=10 total). (**d**) Tumor growth kinetics under different treatment conditions for B16F10 parental model. Statistical significance calculated by two-tailed unpaired t test. Data are presented as mean values +/- SEM. Plots reflect data from 1 experiment (n=5). (**e**) Quantitation of CD8+ T cells per gram tumor. Data are presented as mean values +/- SEM. Data reflect combined data from 2 separate experiments (n=10 total). (**f**) Frequency of PD-1+TCF-1+ cells and (**g**) PD-1+ TIM-3+ in the tumors. Data are presented as mean values +/- SEM. Data reflect combined data from 2 separate experiments (n=10 total). (**h**) Representative flow plots of IFN-γ+ TNF-α+ cells in the tumors. (**i**) Frequency of IFN-γ+ cells in the tumors. Data are presented as mean values +/- SEM. Data reflect combined data from 2 separate experiments (n=10 total). (**j**) Quantitation of the number of IFN-γ. Data are presented as mean values +/- SEM. Data reflect combined data from 2 separate experiments (n=10 total). (**k**) Quantitation of the number of TNF-α+ IFN-γ+ T cells per gram tumor. Data are presented as mean values +/- SEM. Data reflect combined data from 2 separate experiments (n=10 total). Statistical significance calculated by Kruskal-Wallis test, unless otherwise noted. Source data are provided as a Source Data file.

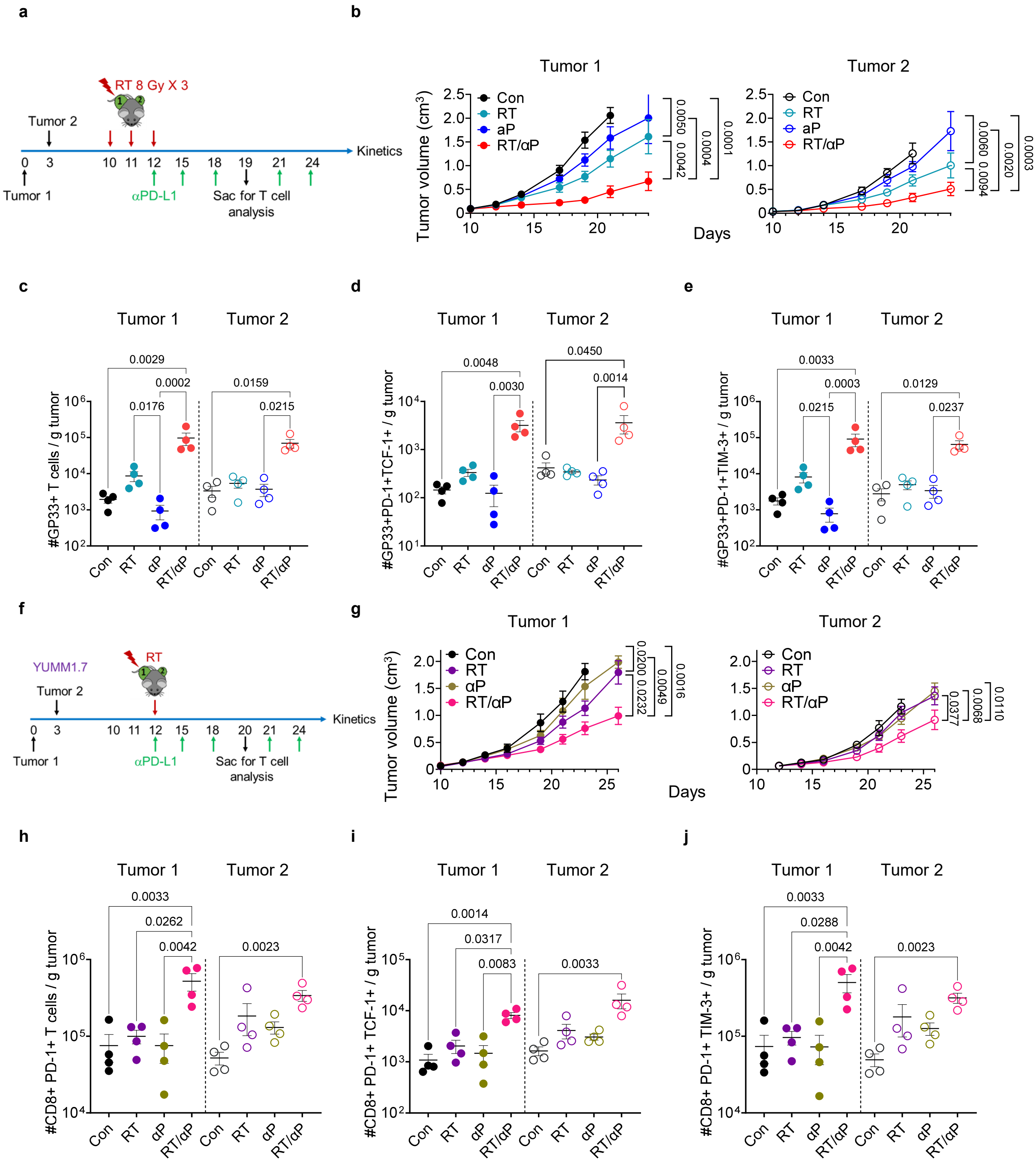

Supplementary Figure 2. RT + αPD-L1 promote an increase in intra-tumoral PD-1+ TCF-1+ and PD-1+ TIM-3+ CD8+ T cells, related to Figure 1. (**a**) Experimental schema with B16F10GP treated with 8Gy x 3 fractions and anti-PD-L1 started the same day as last fraction. (**b**) Tumor growth kinetics under different treatment conditions for B16F10GP. Data are presented as mean values +/- SEM. Plots reflect data from 1 experiment (n=5). Statistical significance calculated by two-tailed unpaired t test. (**c**) Quantitation plots for number of GP33+ T cells per gram tumor. Statistical significance calculated by one-way ANOVA. Data are presented as mean values +/- SEM. Plots reflect data from 1 experiment (n=4). (**d**) Quantitation plots for number of PD-1+ TCF-1+ T cells per gram tumor. Data are presented as mean values +/- SEM. Plots reflect data from 1 experiment (n=4). (**e**) Quantitation plots for number of PD-1+ TIM-3+ T cells per gram tumor. Data are presented as mean values +/- SEM. Plots reflect data from 1 experiment (n=4). (**f**) Experimental schema with YUMM1.7 cell line; 10 Gy x 1 RT was administered. (**g**) Tumor growth kinetics under different treatment conditions for YUMM1.7. Statistical significance calculated by two-tailed unpaired t test. Data are presented as mean values +/- SEM. Plots reflect data from 1 experiment (n=5). (**h**) Quantitation plots for number of PD-1+ T cells per gram tumor. Data are presented as mean values +/- SEM. Plots reflect data from 1 experiment (n=4). (**i**) Quantitation plots for number of PD-1+ TCF-1+ T cells per gram tumor. Data are presented as mean values +/- SEM. Data reflect combined data from 1 experiment (n=4). (**j**) Quantitation plots for number of PD-1+ TIM-3+ T cells per gram tumor. The vast majority of CD8+ PD-1+ T cells in the tumor are TIM-3+ and therefore this plot appears similar to the bulk PD-1+ (h). Data are presented as mean values +/- SEM. Plots reflect data from 1 experiment (n=4). Statistical significance calculated by Kruskal-Wallis test, unless otherwise noted. Source data are provided as a Source Data file.

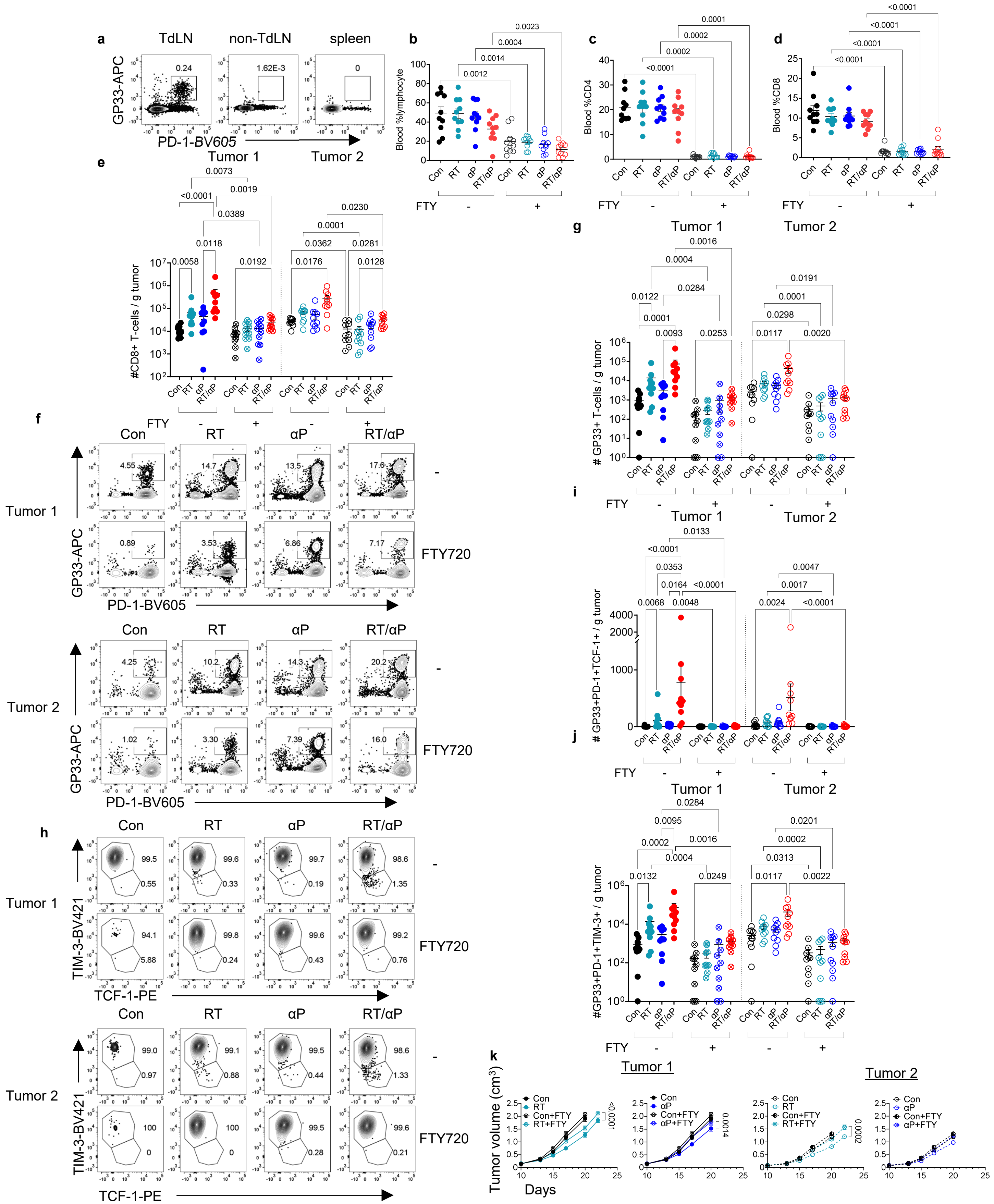

Supplementary Figure 3. FTY720 treatment abolished the increased number of GP33+ T cells and attenuated tumor growth control by RT or anti-PD-L1, related to Figure 2. (**a**) Representative flow plots of GP33+ T cells in different tissues. (**b**) Frequency of lymphocytes, (**c**) CD4+ T cells, and (**d**) CD8+ T cells in blood. Statistical Data are presented as mean values +/- SEM. Data reflect combined data from 2 experiments (n=10). (**e**) Quantitation of CD8+ T cells per gram tumor. Data are presented as mean values +/- SEM. Data reflect combined data from 2 experiments (n=10). (**f**) Representative flow plots of GP33+ T cells in the tumors under different treatment conditions with or without FTY720. (**g**) Quantitation of GP33+ T cells in the tumors under different treatment conditions with or without FTY720. Data are presented as mean values +/- SEM. Data reflect combined data from 2 experiments (n=10). (**h**) Representative flow plots of GP33+ PD-1+ TCF-1+ T cells or TIM-3+ in the tumors under different treatment conditions with or without FTY720. (**i**) Quantitation of GP33+ PD-1+ TCF-1+ T cells per gram tumor and (**j**) PD-1+ TIM-3+ per gram tumor. Data are presented as mean values +/- SEM. Data reflect combined data from 2 experiments (n=10). (**k**) Tumor growth kinetics under different treatment conditions with or without FTY720. Data are presented as mean values +/- SEM. Statistical significance calculated by two-tailed unpaired t test. Data reflect combined data from 2 experiments (n=15). Statistical significance calculated by Kruskal-Wallis test, unless otherwise noted. Source data are provided as a Source Data file.

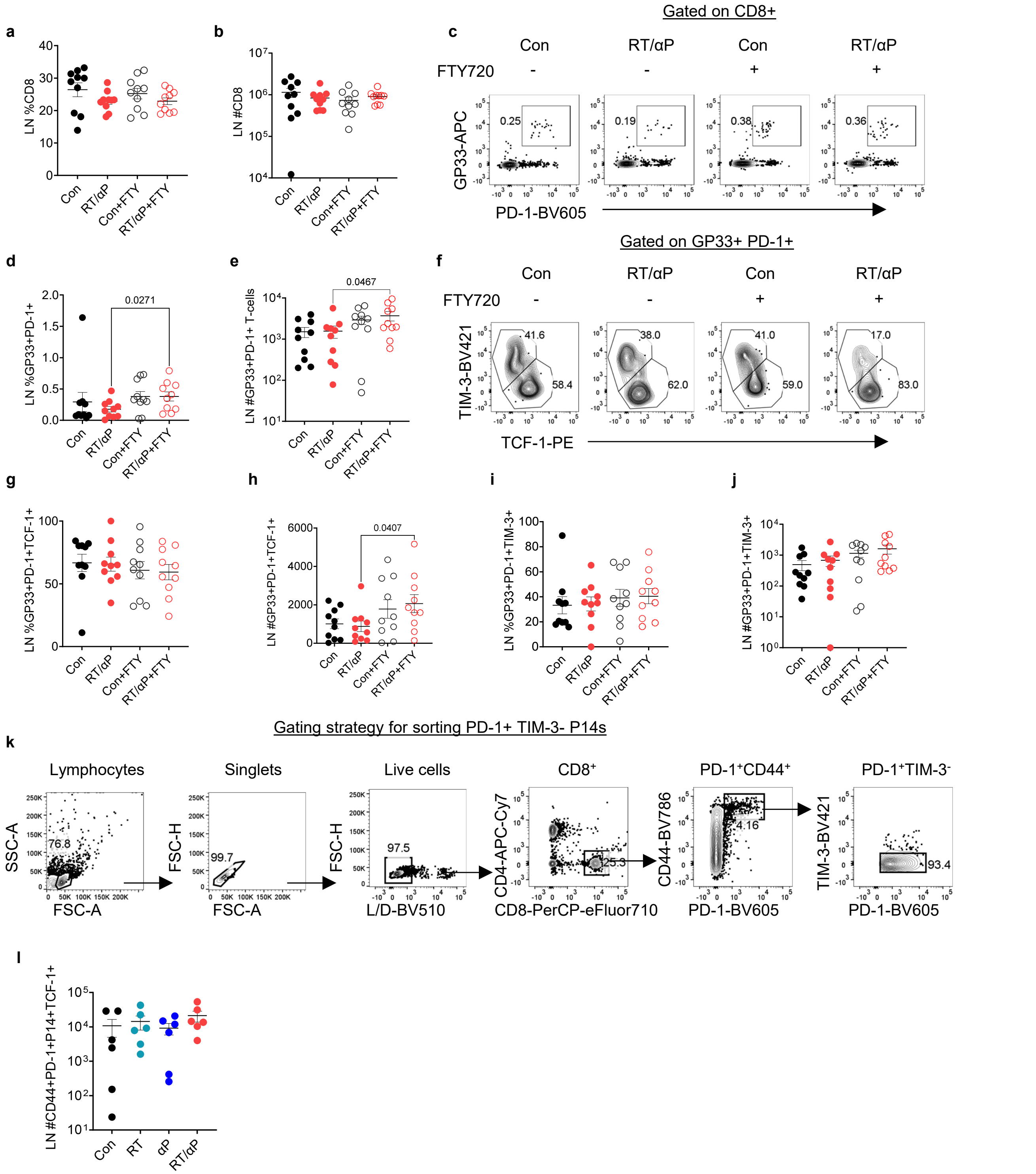

Supplementary Figure 4. Changes of T cells in the TdLN, related to Figure 2 and Figure 3. (**a**) Frequency of CD8+ T cells in the TdLN of tumor 1 under different treatment conditions with or without FTY720. Data are presented as mean values +/- SEM. Plots reflect data from 2 experiments (n=10). (**b**) Number of CD8+ T cells in the TdLN of tumor 1 under different treatment conditions with or without FTY720. Data are presented as mean values +/- SEM. Plots reflect data from 2 experiments (n=10). (**c**) Representative flow plots of GP33+ T cells in the TdLN of tumor 1 under different treatment conditions with or without FTY720. **(d)** Frequency and **(e)** number of GP33+ T cells in the TdLN of tumor 1 under different treatment conditions with or without FTY720. Data are presented as mean values +/- SEM. Plots reflect data from 2 experiments (n=10). (**f**) Representative flow plots of GP33+ PD-1+ TCF-1+ T cells and TIM-3+ in the TdLN of tumor 1 under different treatment conditions with or without FTY720. (**g**) Frequency and (**h**) number of GP33+ PD-1+ TCF-1+ T cells in the TdLN of tumor 1 under different treatment conditions with or without FTY720. Data are presented as mean values +/- SEM. Plots reflect data from 2 experiments (n=10). (**i**) Frequency and (**j**) number of PD-1+ TIM-3+ in the TdLN of tumor 1 under different treatment conditions with or without FTY720. Data are presented as mean values +/- SEM. Plots reflect data from 2 experiments (n=10). (**k**) Gating strategy for sorting PD-1+ CD44+ TIM-3- P14s. (**l**) Quantitation of CD44+ PD-1+ P14+ TCF-1+ cells in the TdLN of tumor 1. Data are presented as mean values +/- SEM. Plots reflect data from 2 experiments (n=6). Statistical significance calculated by Kruskal-Wallis test, unless otherwise noted. Source data are provided as a Source Data file.

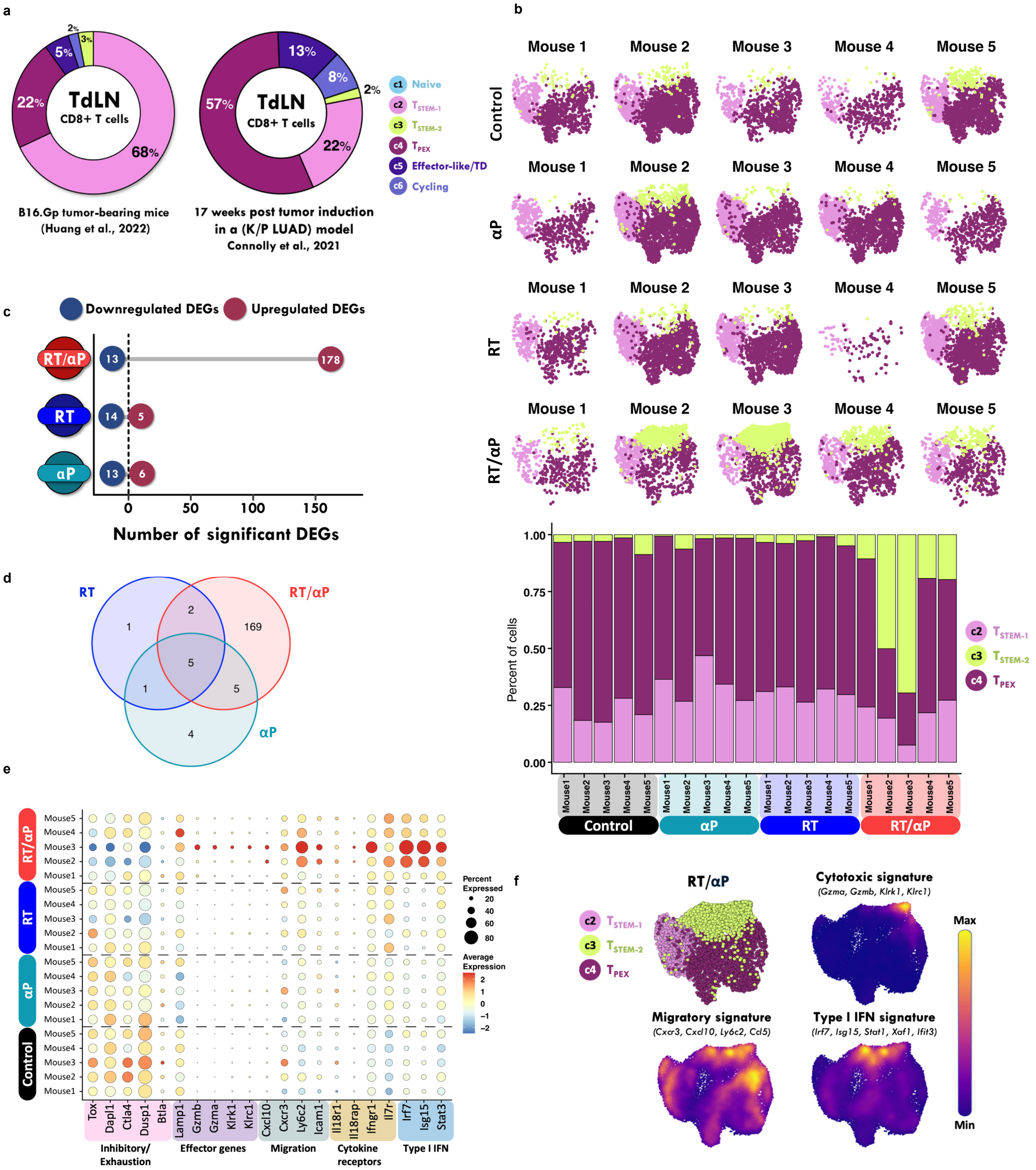

Supplementary Figure 5. RT + αPD-L1 combination therapy induces a novel stem-like subset in the TdLN, related to Figure 4 and Figure 5. (a) Quantitation of different subset frequencies in the TdLN from published data re-analyzed here. **(b)** UMAP and quantitation demonstrating the major *Tcf7*-expressing CD8+ PD-1+ T cell populations in the TdLN under different treatment conditions per mouse. **(c)** Differentially expressed genes (DEGs) relative to untreated controls. **(d)** Venn diagram showing the overlap among DEGs from pairwise comparisons between treatment groups and untreated controls. **(e)** DotPlot showing the expression of marker genes used to distinguish the T_{STEM-2} population per mouse. **(f)** Density plots evaluating the transcriptional signature in the TdLN with RT + αPD-L1 therapy. Source data are provided as a Source Data file and are available on the NCBI Gene Expression Omnibus (GEO) database.

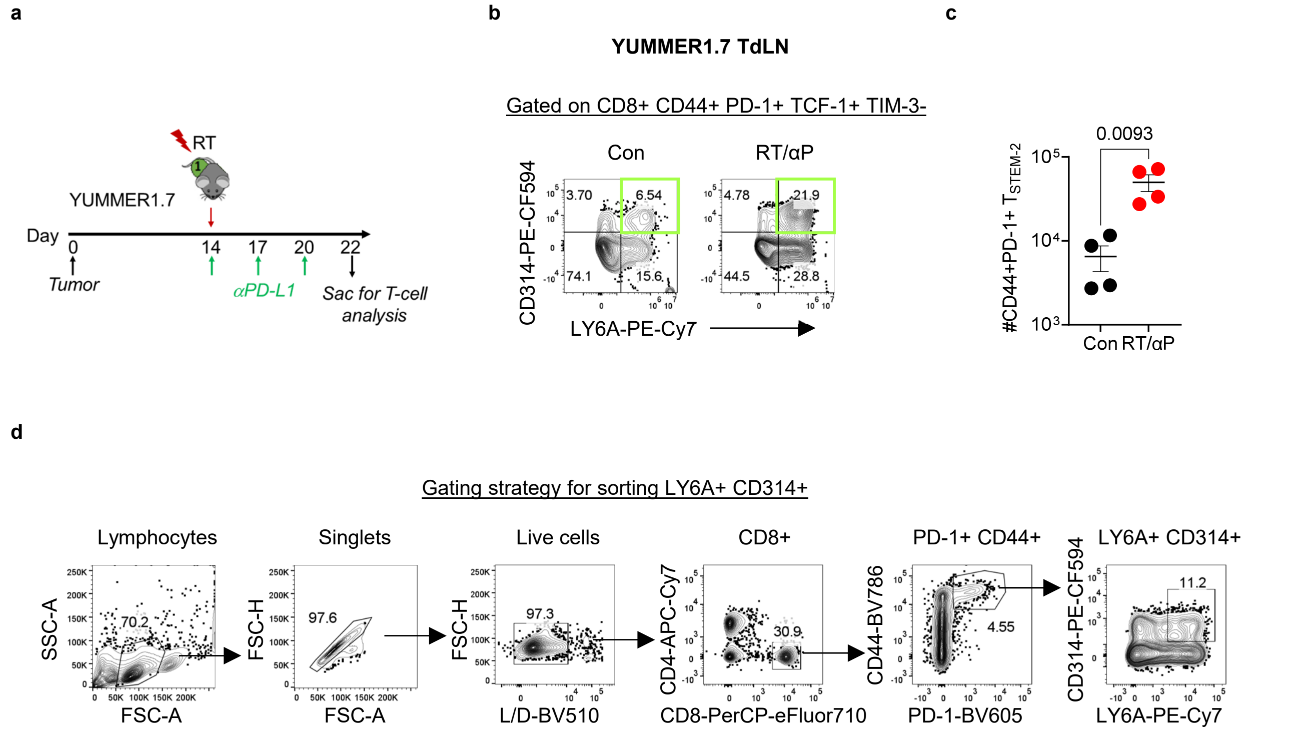

Supplementary Figure 6. RT + αPD-L1 in combination promote T_{STEM-2} population, related to Figure 6. (**a**) Experimental schema with YUMMER1.7 cell line. (**b**) Representative flow plots of LY6A+ CD314+ T cells in the TdLN under different treatment conditions using the Yummer1.7. (**c**) Quantitation of LY6A+ CD314+ cells in the TdLN under different treatment conditions. Statistical significance calculated by two-tailed unpaired t test. Data are presented as mean values +/- SEM. Plots reflect data from 1 experiment (n=4). (**d**) Gating strategy for sorting LY6A+ CD314+ cells. Source data are provided as a Source Data file.

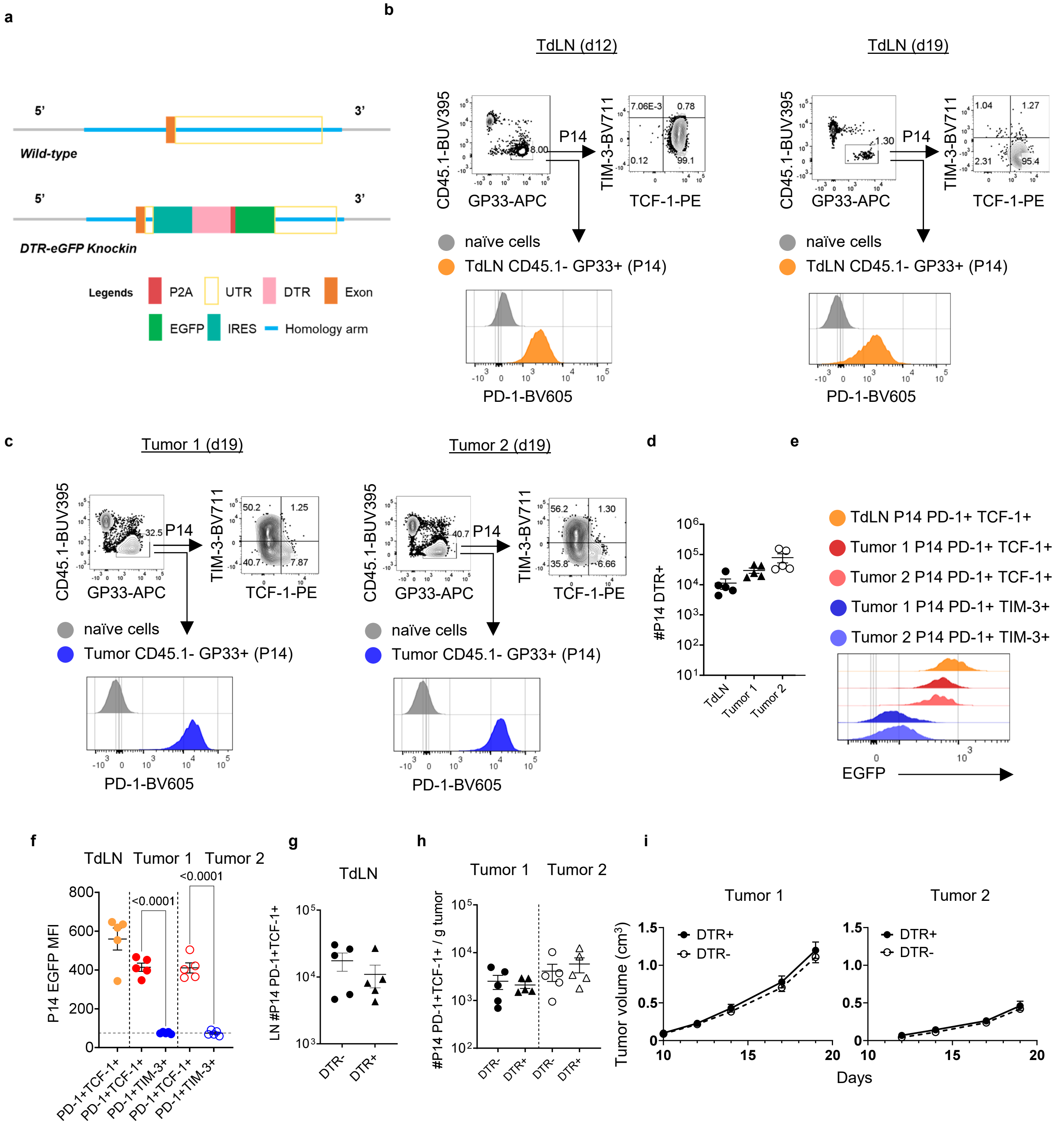

Supplementary Figure 7. Tcf7-DTR-eGFP construct, related to Figure 7. (**a**) Schematic representation of the Tcf7-DTR-eGFP construct. (**b**) Representative flow plots showing the P14 DTR+ T cells differentiating into PD-1+ TCF-1+ in the TdLN of tumor 1 and (**c**) PD-1+ TIM-3+ in the tumors. (**d**) Quantitation of P14 DTR+ cells in the TdLN of tumor 1 and the tumors. Data are presented as mean values +/- SEM. Data shown from a representative experiment n=5 per group. (**e**) Representative flow plots of eGFP MFI in different subsets. (**f**) eGFP MFI quantified by different subsets. Data are presented as mean values +/- SEM. Data shown from a representative experiment n=5 per group. (**g**) Quantitation of P14 DTR+ or DTR- PD-1+ TCF-1+ T cells in the TdLN of tumor 1 or **(h)** tumor for separate recipients. Data are presented as mean values +/- SEM. Data shown from a representative experiment n=5 per group. (**i**) Tumor growth kinetics in mice which received either P14 DTR+ or DTR- T cells. Data are presented as mean values +/- SEM. Data shown from a representative experiment n=5 per group. Statistical significance calculated by two-tailed unpaired t test. Source data are provided as a Source Data file.

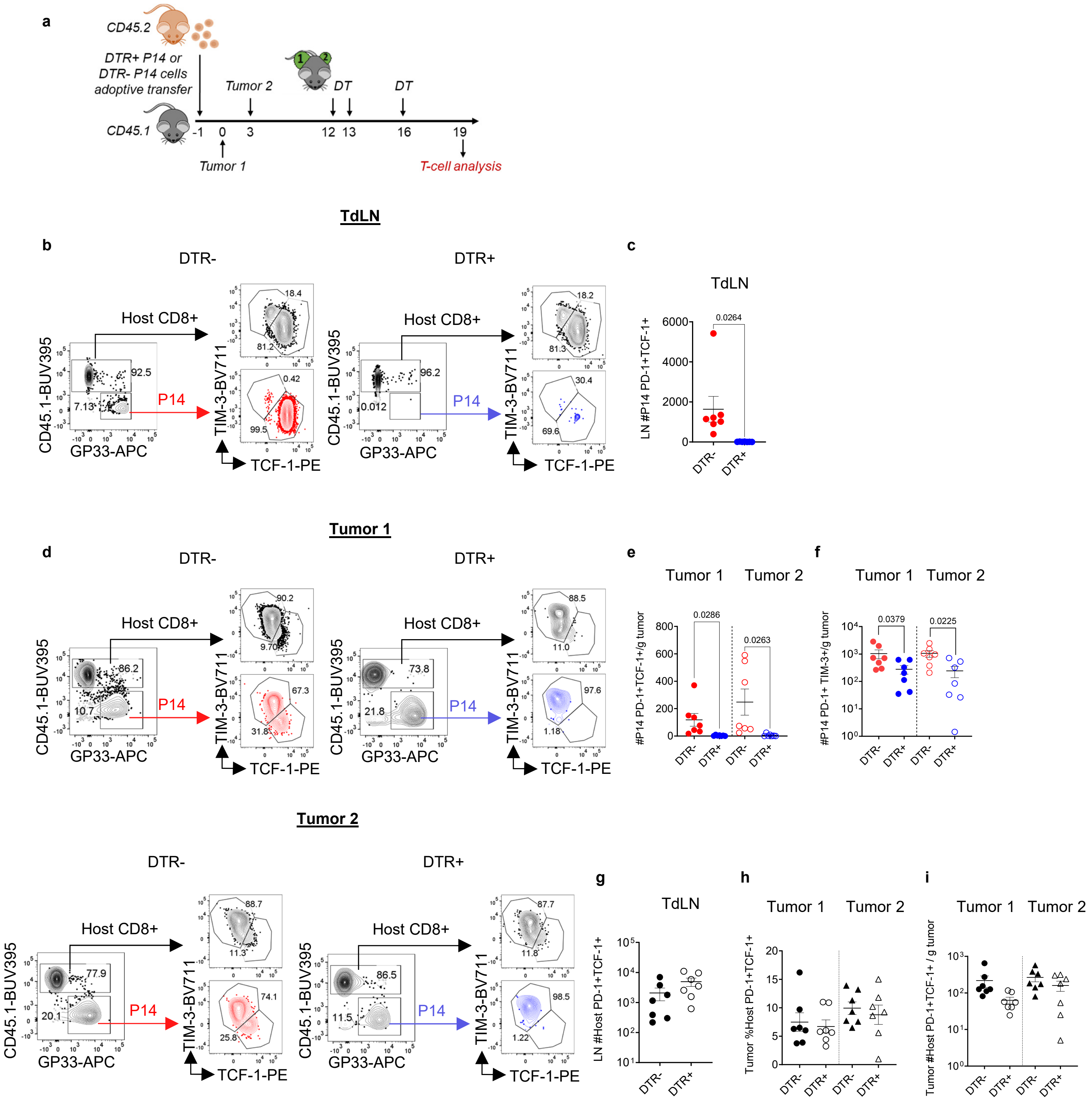

Supplementary Figure 8. P14 Tcf7$^{DTR\text{-}eGFP}$ allows for the specific depletion of TCF-1+ T cells in the TdLN and tumor, related to Figure 7. (**a**) Experimental schema with diphtheria toxin (DT) administration. (**b**) Representative flow plots with gating on the endogenous and transferred P14 T cells in the TdLN of tumor 1 for either DTR- (red) or DTR+ (blue) recipients. (**c**) Quantitation of DTR- or DTR+ P14 PD-1+ TCF-1+ T cells following DT administration in TdLN of tumor 1. Statistical significance calculated by two-tailed unpaired t test. Data are presented as mean values +/- SEM. Data reflect combined data from 2 separate experiments (n=7 total). (**d**) Representative flow plots with gating evaluating transferred and endogenous cells in the tumors. (**e**) Quantitation of P14 PD-1+ TCF-1+ and (**f**) PD-1+ TIM-3+ per gram tumor. Statistical significance calculated by two-tailed unpaired t test. Data are presented as mean values +/- SEM. Data reflect combined data from 2 separate experiments (n=7 total). (**g**) Quantitation of endogenous PD-1+ TCF-1+ T cells. Statistical significance calculated by two-tailed unpaired t test. Data are presented as mean values +/- SEM. Data reflect combined data from 2 separate experiments (n=7 total). (**h**) Quantitation of endogenous PD-1+ TCF-1+ and (**i**) PD-1+ TIM-3+ per gram tumor. Statistical significance calculated by two-tailed unpaired t test. Data are presented as mean values +/- SEM. Data reflect combined data from 2 separate experiments (n=7 total). Source data are provided as a Source Data file.

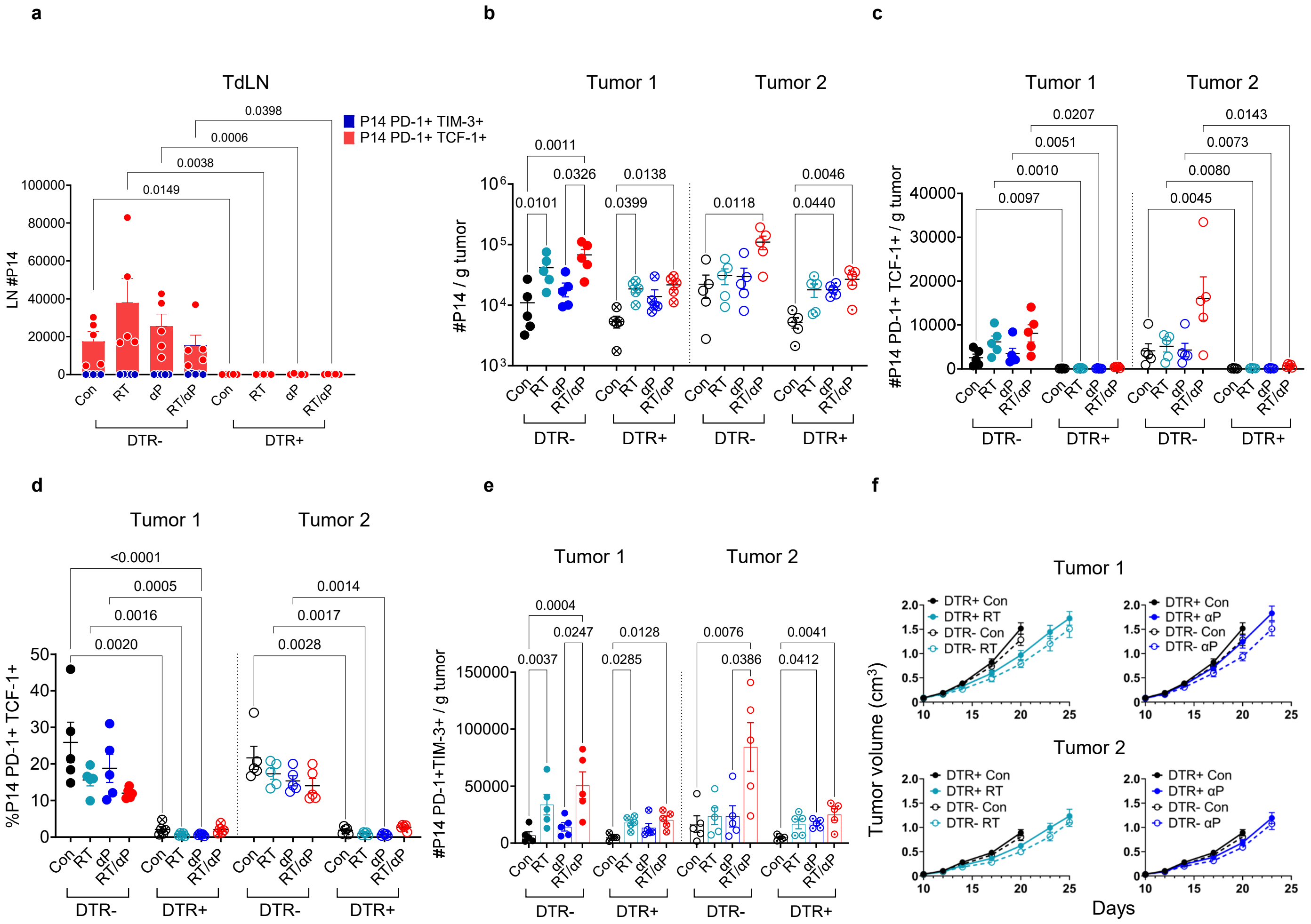

Supplementary Figure 9. Diphtheria toxin administration depleted TCF-1+ T cells in the tumors and TdLN, related to Figure 7. (**a**) Quantitation of PD-1+ TCF-1+ T cells and PD-1+ TIM-3+ in the TdLN of tumor 1 under different treatment conditions with DTR+ or DTR- P14 transfer. Data are presented as mean values +/- SEM. Data shown from a representative experiment n=5 per group. (**b**) Quantitation of P14 cells per gram tumor. Data are presented as mean values +/- SEM. Data shown from a representative experiment n=5 per group. (**c**) Quantitation of PD-1+ TCF-1+ T cell number per gram tumor. Data are presented as mean values +/- SEM. Data shown from a representative experiment n=5 per group. (**d**) PD-1+ TCF-1+ T cell frequency under different treatment conditions in P14 DTR+ or DTR- recipients. Data are presented as mean values +/- SEM. Data shown from a representative experiment n=5 per group. (**e**) Number of PD-1+ TIM-3+ T cells in the tumors under different treatment conditions in P14 DTR+ or DTR- recipients. Data are presented as mean values +/- SEM. Data shown from a representative experiment n=5 per group. (**f**) Tumor growth kinetics under different treatment conditions in P14 DTR+ or DTR- recipients. Data are presented as mean values +/- SEM. Statistical significance calculated by two-tailed unpaired t test. Data shown from two separate experiments. n=10 per group. Statistical significance calculated by Kruskal-Wallis test, unless otherwise noted. Source data are provided as a Source Data file.
