# Supplementary material for: Combination radiation and αPD-L1 enhance tumor control by stimulating CD8+ PD-1+ TCF-1+ T cells in the tumor-draining lymph node
Source: Nat Commun. 2025 Apr 14;16:3522. doi: 10.1038/s41467-025-58510-1 (PMC11997041; doi:10.1038/s41467-025-58510-1)
Supplement: Supplementary file 2 — Description of Additional Supplementary Files [file 41467_2025_58510_MOESM2_ESM.pdf]

Description of Additional Supplementary Files

Supplementary Data 1: Key Resources Table
