# Supplementary material for: Combination radiation and αPD-L1 enhance tumor control by stimulating CD8+ PD-1+ TCF-1+ T cells in the tumor-draining lymph node
Source: Nat Commun. 2025 Apr 14;16:3522. doi: 10.1038/s41467-025-58510-1 (PMC11997041; doi:10.1038/s41467-025-58510-1)
Supplement: Supplementary file 3 — Supplementary Data 1 [file 41467_2025_58510_MOESM3_ESM.docx]

**Key Resources Table**

| **Reagent or resource** | **Source** | **Cat#** |
| --- | --- | --- |
| **Antibodies and tetramer (dilution)** | | |
| Anti-Mouse CD45.1 - BUV395 (1:100) | BD Biosciences | Cat#565212; RRID:AB_2722493 |
| Anti-Mouse CD45.2 - BUV563 (1:100) | BD Biosciences | Cat#741273; RRID:AB_2870814 |
| Anti-Mouse CD8a - PerCP-eFluor 710 (1:100) | eBioscience | Cat#46-0081-82; RRID:AB_1834433 |
| Anti-Mouse CD4 - PE/Cy7 (1:100) | BioLegend | Cat#100422; RRID:AB_312707 |
| Anti-Mouse CD4 - BV480 (1:100) | BD Biosciences | Cat#746475; RRID:AB_2743777 |
| H-2Db-gp33-tetramer – APC (1:100) | NIH Tetramer Core Facility | N/A |
| Anti-Mouse Tim-3 - BV711 (1:100) | BioLegend | Cat#119727; RRID:AB_2716208 |
| Anti-Mouse Tim-3 - BV421 (1:100) | BioLegend | Cat#119723; RRID:AB_2716908 |
| Anti-Mouse PD-1 - BV605 (1:100) | BioLegend | Cat#135220; RRID:AB_2562616 |
| Anti-Mouse Ly-6A/E - BV510 (1:100) | BD Biosciences | Cat#744323; RRID:AB_2742151 |
| Anti-Mouse Ly-6A/E - PE-Cy7 (1:200) | BioLegend | Cat#122514; RRID:AB_756199 |
| Anti-Mouse TCF-7/TCF-1 – PE (1:100) | BD Biosciences | Cat#564217; RRID:AB_2687845 |
| Anti-Human Granzyme B - Alexa Fluor 700 (1:100) | BD Biosciences | Cat#560213; RRID:AB_1645453 |
| Anti-Human Granzyme B - BV510 (1:100) | BD Biosciences | Cat#563388; RRID:AB_2738174 |
| Anti-Mouse CD62L - BUV737 (1:200) | BD Biosciences | Cat#612833; RRID:AB_2870155 |
| Anti-Mouse IFN gamma – APC (1:100) | eBioscience | Cat#17-7311-82; RRID:AB_469504 |
| Anti-Mouse TNF – PE | BD Biosciences | Cat#554419; RRID:AB_395380 |
| Anti-Mouse CD314 - PE-CF594 (1:200) | BD Biosciences | Cat#562614; RRID:AB_2737677 |
| Anti-Mouse TOX - Alexa Fluor 647 (1:100) | BD Biosciences | Cat#568356 |
| Anti-Mouse CCRL2 - RB545 (1:100) | BD Biosciences | Cat#756534 |
| Anti-Mouse IFITM3 - CL488 (1:100) | Proteintech | Cat# CL488-11714; RRID:2919038 |
| TotalSeq™-C0301 anti-mouse Hashtag 1 Antibody (1:100) | BioLegend | Cat# 155861; RRID:AB_2800693 |
| TotalSeq™-C0302 anti-mouse Hashtag 2 Antibody (1:100) | BioLegend | Cat# 155863; RRID:AB_2800694 |
| TotalSeq™-C0303 anti-mouse Hashtag 3 Antibody (1:100) | BioLegend | Cat# 155865; RRID:AB_2800695 |
| TotalSeq™-C0304 anti-mouse Hashtag 4 Antibody (1:100) | BioLegend | Cat# 155867; RRID:AB_2800696 |
| TotalSeq™-C0305 anti-mouse Hashtag 5 Antibody (1:100) | BioLegend | Cat# 155869; RRID:AB_2800697 |
| **Chemicals, peptides, and recombinant proteins** | | |
| Diphtheria Toxin (DT) | Sigma-Aldrich | Cat#D0564 |
| FTY720 | Enzo Life Sciences | Cat#BML-SL233-0025 |
| Ghost Dye Red 780 | Tonbo Biosciences | Cat#13-0865-T100 |
| Gp33 | AnaSpec Inc | Cat#AS-61296 |
| Gp276 | AnaSpec Inc | Cat#AS-62539 |
| Dulbecco’s Modified Eagle’s Medium | Corning | Cat#15-017-CV |
| Penicillin-Streptomycin | Gibco | Cat#15-140-122 |
| L-Glutamine | Gibco | Cat#25030081 |
| **Critical commercial assays** | | |
| eBioscience Foxp3 / Transcription Factor Staining Buffer Set | Invitrogen | Cat#00-5523-00 |
| LIVE/DEAD™ Fixable Aqua Dead Cell Stain Kit | Invitrogen | Cat#L34957 |
| Fixation and Permeabilization Solution | BD Biosciences | Cat#554722 |
| Perm/Wash Buffer | BD Biosciences | Cat#554723 |
| AllPrep DNA/RNA Micro Kit | QIAGEN | Cat#80284 |
| QIAshredder | QIAGEN | Cat#79654 |
| **Deposited data** | | |
| RNA-Seq data | This study | N/A |
| TCR-Seq data | This study | N/A |
| **Experimental models: Cell lines** | | |
| B16F10GP (derived from B16-F10) | This study | N/A |
| B16-F10 | ATCC | RRID:CVCL_0159 |
| YUMM1.7 | Paulos lab | RRID:CVCL_JK16 |
| YUMMER1.7 | Paulos lab | RRID:CVCL_A2AX |
| **Experimental models: Organisms/strains** | | |
| Mouse: C57BL/6J (B6 CD45.2) | Jackson Laboratory | Straining#000664; RRID:IMSR_JAX:000664 |
| Mouse: B6.SJL-Ptprc <a> (B6 CD45.1) | Jackson Laboratory | Strain#002014; RRID:IMSR_JAX:002014 |
| Mouse: B6.Tg(Tcf7^EGFP-DTR^) | This study | N/A |
| Mouse: B6.Cg-Tcratm1Mom  Tg(TcrLCMV)327Sdz/TacMmjax | Jackson Laboratory | Strain #037394-JAX; RRID:MMRRC_037394-JAX |
| **Software and algorithms** | | |
| Prism (v9) | GraphPad | RRID:SCR_002798 |
| FlowJo (v10.8.0) | BD | RRID:SCR_008520 |
| R (v4.2.1) | <http://www.r-project.org/> | RRID:SCR_001905 |
| Seurat package (v4.1.1) | <https://satijalab.org/seurat/> | RRID:SCR_016341 |
| Cell Ranger | <https://www.10xgenomics.com/support/software/cell-ranger/latest> | RRID:SCR_017344 |
